# Green turtles (*Chelonia mydas*) foraging at Arvoredo Island in Southern Brazil: Genetic characterization and mixed stock analysis through mtDNA control region haplotypes

**DOI:** 10.1590/S1415-47572009000300027

**Published:** 2009-09-01

**Authors:** Maíra Carneiro Proietti, Paula Lara-Ruiz, Júlia Wiener Reisser, Luciano da Silva Pinto, Odir Antonio Dellagostin, Luis Fernando Marins

**Affiliations:** Programa de Pós-Graduação em Oceanografia Biológica, Universidade Federal do Rio Grande, Rio Grande, RSBrazil; 2 Grupo de Identificación, Unidad de Especies Silvestres, Instituto de Genética, Universidad Nacional de Colombia, Ciudad Universitaria, BogotáColombia; 3 Centro de Biotecnologia, Universidade Federal de Pelotas, Campus Universitário, Pelotas, RSBrazil; 4 Departamento de Ciências Fisiológicas, Universidade Federal do Rio Grande, Rio Grande, RSBrazil

**Keywords:** foraging grounds, genetic diversity, green turtle, mtDNA haplotypes, natal origins

## Abstract

We analyzed mtDNA control region sequences of green turtles (*Chelonia mydas*) from Arvoredo Island, a foraging ground in southern Brazil, and identified eight haplotypes. Of these, CM-A8 (64%) and CM-A5 (22%) were dominant, the remainder presenting low frequencies (< 5%). Haplotype (*h*) and nucleotide (π) diversities were 0.5570 ± 0.0697 and 0.0021 ± 0.0016, respectively. Exact tests of differentiation and AMOVA Φ_ST_ pairwise values between the study area and eight other Atlantic foraging grounds revealed significant differences in most areas, except Ubatuba and Rocas/Noronha, in Brazil (p > 0.05). Mixed Stock Analysis, incorporating eleven Atlantic and one Mediterranean rookery as possible sources of individuals, indicated Ascension and Aves islands as the main contributing stocks to the Arvoredo aggregation (68.01% and 22.96%, respectively). These results demonstrate the extensive relationships between Arvoredo Island and other Atlantic foraging and breeding areas. Such an understanding provides a framework for establishing adequate management and conservation strategies for this endangered species.

The green turtle (*Chelonia mydas*) is a marine reptile of worldwide tropical and subtropical distribution, currently classified by the World Conservation Unit as endangered (IUCN, 2007). These animals present complex and long life histories, together with a highly migratory behaviour (Meylan, 1995; Godley *et al.*, 2003). Due to the large temporal and spatial scales involved, various aspects of their life cycle are quite difficult to elucidate by conventional approaches, and must be solved by using indirect research methods, such as molecular genetics (Avise, 2007; Bowen and Karl, 2007).

Mitochondrial DNA (mtDNA) control region studies have been increasingly applied to marine turtles, whereby the development of genetic tags for these animals has contributed to the acquisition of valuable data on their molecular evolution, population structure, reproductive behavior and migration ecology, besides providing a foundation for conservation and management strategies (Moritz, 1994; Avise, 2007; Bowen and Karl, 2007). In this context, green turtles have emerged as model organisms for such studies (Avise, 2007). These animals forage in “mixed stocks” composed of individuals from several cohorts and from various nesting beaches (rookeries) which aggregate at feeding grounds (Bass and Witzell, 2000; Bass *et al.*, 2006; Avise, 2007; Bowen and Karl, 2007). Due to the phylopatric behaviour of the females of this species, nesting assemblages are genetically structured in terms of maternally-inherited characters, thereby permitting the evaluation of the natal origins of individuals found in mixed aggregations (Bowen, 1995; Bowen and Karl, 2007). The assessment of the genetic composition of mixed aggregations is currently one of the research priorities for this species (Formia *et al.*, 2006). This, together with the determination of the relationships among foraging and breeding populations of sea turtles, are essential for constituting secure guide lines in the development of successful conservation strategies for these endangered animals.

Sampling was undertaken at Arvoredo Island, located within the Arvoredo Marine Biological Reserve (27° 17' S and 48° 22' W), in July 2005, January-February 2006 and July 2006, at five different sites located on the western and northern parts of the island (Figure 1). Tissue samples were obtained from the flippers of 49 juvenile green turtles hand-captured through free and SCUBA dives, by using 5 mm disposable biopsy punches. The samples were then conserved in absolute ethanol and kept at -20 °C. Curved carapace length and the weight of sampled individuals ranged from 35 to 72.5 cm (mean 52 cm) and 7.5 to 45 kg (mean 19.9 kg), respectively.

DNA extraction was performed through the standard phenol:chlorophorm method with precipitation in absolute ethanol (Hillis *et al.*, 1996). Control region fragments were amplified via polymerase chain reactions (PCR) using the primers LTCM1 and HDCM1 (Allard *et al.*, 1994), under the following conditions: initial denaturation of 1 min at 94 °C, 35 cycles of 30 s at 94 °C, 1 min at 50 °C, 1 min at 72 °C, and a final 5 min extension at 72 °C. Products were purified using Illustra GFX purification kits (GE Healthcare, U.S.A.), and sequenced in both directions using DYEnamic ET dye terminator kits in a MegaBACE 500 DNA sequencer (GE Healthcare, U.S.A.).

Sequences (491 bp) were aligned by means of Clustal X 1.83 software (Thompson *et al.*, 1997), and haplotypes classified according to the Archie Carr Center for Sea Turtle Research online genetic bank (Florida University). A minimum spanning network demonstrating relationships among haplotypes was set up using TCS 1.3 software (Clement *et al.*, 2000). Exact tests of differentiation between Arvoredo Island and other Atlantic foraging grounds were carried out with Arlequin 3.11 (Excoffier *et al.*, 2005), using Markov Chain Monte Carlo (MCMC) of 10000 permutations with 1000 dememorization steps. Pairwise Φ-statistics (Φ_ST_, which summarizes the degree of differentiation between populations) were checked through Analysis of Molecular Variance (AMOVA) conducted with 10000 permutations with Arlequin 3.11, according to the Tamura-Nei model of nucleotide substitution. The Brazilian foraging grounds included in these analyses for comparison were Ubatuba (SP), Almofala (CE) (Naro-Maciel *et al.*, 2007), Rocas Atoll (RN) and Fernando de Noronha (PE) (Bjorndal *et al.*, 2006). The latter two were grouped into one single unit for all analyses, due to geographic proximity (c.a. 150 km) and small sample size, being hereafter referred to as Rocas/Noronha. Nicaragua (Bass *et al.*, 1998), Barbados (Luke *et al.*, 2004), Bahamas (Lahanas *et al.*, 1998), Florida (Bass and Witzell 2000) and North Carolina (Bass *et al.*, 2006), in the Caribbean and North Atlantic, were also included for comparison. Structuring between foraging areas grouped into North and South Atlantic aggregations was checked through AMOVA.

Probable natal origins were defined through Mixed Stock Analysis (MSA) employing Bayes software (Pella and Masuda, 2001), and considering equal prior probabilities assigned to each source. Source populations employed as possible contributors to the Arvoredo Island group correspond to all the Atlantic and Mediterranean rookeries described in literature by Bjorndal *et al.* (2005, 2006), Formia *et al.* (2006, 2007), Encalada *et al.* (1996) and Kaska (2000), namely, Trindade Island and Rocas/Noronha (Brazil), Ascension Island (United Kingdom), Poilão (Guinea Bissau), Bioko Island (Equatorial Guinea), São Tomé (Democratic Republic of São Tomé and Príncipe), Aves Island (Venezuela), Matapica (Surinam), Quintana Roo (Mexico), Tortuguero (Costa Rica), Florida (United States) and Lara Bay (Cyprus). Principe (Democratic Republic of São Tomé and Príncipe) was excluded from this analysis due to the small size of the sample.

We encountered eight polymorphic sites which defined eight previously described Atlantic Ocean haplotypes. The predominant haplotype was CM-A8 (64%), commonly found in South Atlantic rookeries, followed by CM-A5 (22%), mainly found in the Costa Rica, Surinam and Aves Island rookeries. The remaining haplotypes were relatively rare, with less than 5% frequency. To date, CM-A9 (2%), CM-A24 (4%) and CM-A32 (2%) have only been observed in the South Atlantic rookeries of Rocas Atoll, Trindade and Ascension Island, whereas CM-A10 (2%) has been found in Rocas Atoll and Ascension Island. CM-A39 (2%), previously unregistered in foraging areas, and CM-A45 (2%), with only one register in feeding grounds, have been described only in animals from the Ascension Island rookery. Haplotypes were distinguished by a maximum of two variations, as shown in the Minimum Spanning Network (Figure 2).

**Figure 1:**
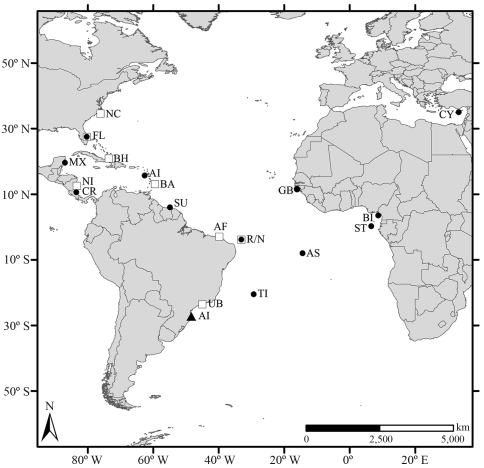
Location of Arvoredo Island (AI - triangle) and other foraging and nesting areas used for comparison and Mixed Stock Analysis. Abbreviations for foraging grounds (squares) are: UB (Ubatuba), R/N (Rocas/Noronha), AF (Almofala), BA (Barbados), BH (Bahamas), NI (Nicaragua), FL (Florida) and NC (North Carolina). Abbreviations for nesting areas (circles) are: TI (Trindade Island), R/N (Rocas/Noronha), AS (Ascension Island), GB (Guinea Bissau), BI (Bioko), ST (São Tomé), AV (Aves Island), SU (Surinam), MX (Mexico), CR (Costa Rica), FL (Florida) and CY (Cyprus).

Haplotype (*h*) and nucleotide (π) diversity estimates encountered for the study area were 0.5570 ± 0.0697 and 0.0021 ± 0.0016, respectively. Diversity estimates for Arvoredo Island and other Atlantic foraging grounds are listed in Table 1. Exact tests of differentiation based on haplotype frequencies demonstrated general differentiation among all feeding areas (p = 0.000). According to these tests, Arvoredo Island was significantly different from most foraging areas, with the exception of Ubatuba and Rocas/Noronha in Brazil (p = 0.4776 and 0.3077, respectively). Similar results were inferred from AMOVA (p = 0.1292 and 0.6261). By grouping foraging aggregations into North and South Atlantic and using AMOVA, strong structuring was revealed (Φ_ST_ = 0.6913 p < 0.01). From MSA, it was indicated that Ascension and Aves Islands are the main contributors to the Arvoredo aggregation, with lesser contributions from Guinea Bissau and Trindade Island, as shown in Table 2.

High CM-A8 frequency in the study area is in accordance with the predominance of this haplotype in various nesting and feeding areas in the Atlantic, and is consistent with the suggestion of it being the closest relative to an ancestral haplotype in the Atlantic basin. Haplotype CM-A5 was the second most frequent, as was noted in other south Atlantic feeding grounds, and in accordance with its high frequency in large Caribbean rookeries (Bjorndal *et al.*, 2005, 2006; Formia *et al.*, 2006, 2007; Naro-Maciel *et al.*, 2007). Elevated *h* values are found in most green turtle foraging areas, as expected when considering that these aggregations are composed of mixed stocks (Bass and Witzell, 2000). Low π values were also expected due to the slight variation observed between haplotypes.

The distribution of haplotypes among foraging grounds is apparently non-random, with significant differentiation among individual areas and strong structuring between North and South Atlantic aggregations. The life history patterns of sea turtles may account for such structuring, with the pelagic stage determining the areas to which these animals will recruit, possibly at the whim of ocean currents (Musick and Limpus, 1997; Luschi *et al.*, 2003). Arvoredo Island was not significantly different from the closest genetically-described southwestern Atlantic foraging ground, Ubatuba (ca. 755 km), thereby indicating that foraging areas can present similarity in mtDNA at small spatial scales. Such a hypothesis is corroborated by Almofala, the most distant southwestern Atlantic foraging ground from Arvoredo Island (ca. 3800 km), being significantly different from the study area. This difference could also be due to its proximity to the Caribbean region, with its strong structuring within the Atlantic Ocean (Bass *et al.* 2006). The similarity observed between relatively close feeding grounds could possibly be attributed to movements along the coast, which may be influenced by factors such as current intensity, variations in temperature, food availability and continuous recruitment to coastal zones (Bass *et al.*, 2006). Despite many animals presenting high fidelity to foraging areas, it is known that non-reproductive costal movements of juvenile green turtles may occur (Godley *et al.*, 2003; Bass *et al.*, 2006), the geographic nearness of the areas and major coastal currents also possibly constituting important factors in these movements. Green turtles present at least short-term fidelity to Arvoredo Island, as demonstrated by various recaptures over a three-year study period (Reisser *et al.*, 2008). Nevertheless, one animal tagged in the area was encountered six months later by members of Project Tamar-ICMBio, stranded at Caraguatatuba in São Paulo state, over 700 km away, thus giving evidence of non-reproductive migration in coastal waters. Migration between São Paulo and southern Brazil has also been observed by Marcovaldi *et al.* (2000), in which a green turtle, initially tagged at Ubatuba, was recaptured three months later in Bombinhas, SC, only 10 km from Arvoredo Island.

**Figure 2:**
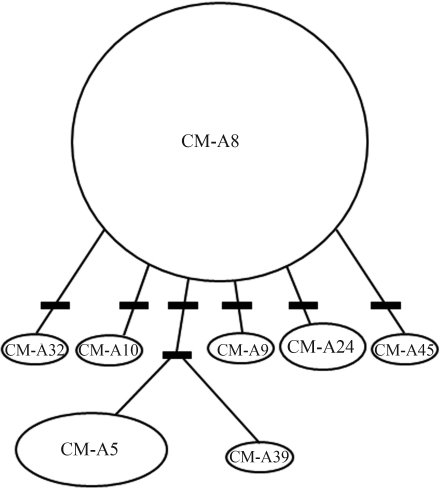
Minimum spanning network of mtDNA control region relationships encountered at Arvoredo Island. Hash lines represent 1 basepair substitution between haplotypes.

As was the case for other south Atlantic foraging areas (Bjorndal *et al.*, 2006; Naro- Maciel *et al.*, 2007), the main stock contributing to the Arvoredo aggregation belongs to Ascension Island. Green turtle movements between Ascension and Brazil have often been noted through mark-recapture and telemetry studies (Meylan, 1995, Luschi *et al.*, 1998, Hays *et al.*, 2002). The large nesting population (the second largest in the Atlantic, with approximately 3800 females nesting annually; Broderick *et al.*, 2006) and favorable ocean currents are the most probable explanations for such a high contribution. The second largest contributor was Aves Island, although there is a lack of tagging evidence on migrations to-and-from Brazil. However, the relatively large rookery size (300-500 females nesting annually; Seminoff, 2002), and the strong link between Caribbean rookeries and Brazilian foraging grounds, as demonstrated by Lima *et al.*, (2008), give support to this conclusion. The connection between African rookeries and Brazilian foraging grounds is not evident, possibly due to the limited number of studies dealing with the African continent. Estimates inferred from MSA indicated that African contributions as a whole to Arvoredo Island were generally low, although those from Guinea Bissau and Bioko were relatively high compared to other African nesting areas. Naro-Maciel *et al.* (2007) also observed a relatively high contribution from Guinea Bissau to Ubatuba. This could be a consequence of the fixed characteristics of this area for the commonly found haplotype CM-08 (Formia *et al.*, 2006), which could have affected MSA estimates. Bioko also presents a high frequency of haplotype CM-08 (90%), also possibly interfering with the analysis. The contribution from Trindade Island is apparently underestimated when considering that this island supports the largest nesting area in Brazil (approximately 300-400 females during the last nesting season - Soares LS, personal communication to PLR), and is the nearest rookery to the study area (*ca*. 2100 km). Furthermore, numerous recaptures of green turtles tagged in this area have been registered along the Brazilian coast (Marcovaldi *et al.*, 2000). Low estimated contributions from Trindade Island have also been registered for the previously cited mixed stocks described in Brazil (Almofala, Ubatuba, Rocas/Noronha). However, in a recent study by Bolker *et al.* (2007), a ‘many-to-many' MSA approach with the incorporation of multiple mixed stocks gave evidence of higher contributions from Trindade Island to northeastern Brazil than those previously published. This could corroborate the hypothesis that Trindade's contribution to the study area is underestimated. Nonetheless, further investigation is necessary to clarify this.

The assumption that all sources and all mixtures are well described is a great problem with MSA, since this is not always the case. The presence of foraging ground haplotypes which have not been described at nesting areas clearly indicates that some rookeries may be inadequately described or not even at all, as was noted by Bass *et al.* (2006), Formia *et al.* (2007) and Naro-Maciel *et al.* (2007). Furthermore, haplotypes being encountered in rookeries but not in foraging areas demonstrates insufficient research at feeding grounds. Therefore, this analysis should be interpreted together with all available evidence (*i.e.* demographic, ecological, and molecular), in order to reach conclusive information on the life history patterns of sea turtles.

Describing the genetic characteristics of juvenile green turtle foraging grounds and defining their relationship with other feeding and breeding grounds provide a framework for successfully conserving and managing this species. The extensive Brazilian coastline and oceanic islands harbor countless foraging grounds, besides three rookeries of which two are relatively large, thereby urging investigation and protection for conservation purposes. Impacts affecting foraging areas may also influence distant rookeries. Thus, the protection of feeding zones could be a big step towards the protection of their contributing stocks. The distribution and migrations of green turtles surpass national boundaries, wherefore urging national and international efforts and cooperation is essential for assuring the survival of this species.

## Figures and Tables

**Table 1:** Haplotype (*h*) and nucleotide (π) diversity estimates ± standard deviations for all compared foraging aggregations.

Foraging ground	Haplotypes	*h*	π	Sample size
Arvoredo Island	8	0.5570 ± 0.0697	0.0021 ± 0.0016	49
Ubatuba^a^	10	0.4460 ± 0.0556	0.0020 ± 0.0015	113
Rocas/Noronha^b^	6	0.5887 ± 0.0911	0.0019 ± 0.0015	32
Almofala^a^	13	0.7168 ± 0.0306	0.0067 ± 0.0039	117
Barbados^c^	8	0.7734 ± 0.0276	0.0105 ± 0.0057	60
Bahamas^d^	6	0.3703 ± 0.0650	0.0066 ± 0.0038	79
Nicaragua^e^	2	0.1831 ± 0.0621	0.0039 ± 0.0025	60
Florida^f^	6	0.4855 ± 0.0668	0.0032 ± 0.0021	62
North Carolina^g^	8	0.6778 ± 0.0310	0.0052 ± 0.0031	106
Average	7	0.5334	0.0040	68

^a^Naro-Maciel *et al.* 2007. ^b^Bjorndal *et al.* 2006. ^c^Luke *et al.* 1994. ^d^Lahanas *et al.* 1998. ^e^Bass *et al.* 1998. ^f^Bass and Witzell 2000. ^g^Bass *et al.* 2006.

**Table 2:** Mixed stock analysis based on Bayesian methods considering equal priors, with mean, standard deviation (S.D.), 2.5% quantile, median and 97.5% quantile.

Stock	Mean	S.D.	2.5%	Median	97.5%
Trindade Island^a^	0.0218	0.0535	0.0000	0.0001	0.1852
Rocas/Noronha^a^	0.0161	0.0471	0.0000	0.0000	0.1700
Ascension Island^b, c, d^	0.6801	0.1171	0.3869	0.7029	0.8407
Guinea Bissau^c^	0.0197	0.0542	0.0000	0.0000	0.1948
Bioko^c^	0.0174	0.0504	0.0000	0.0000	0.1710
São Tomé^c^	0.0062	0.0220	0.0000	0.0000	0.0663
Aves Island^d^	0.2296	0.0597	0.1257	0.2257	0.3592
Surinam^d^	0.0019	0.0064	0.0000	0.0000	0.0199
Mexico^d^	0.0019	0.0064	0.0000	0.0000	0.0196
Costa Rica^e^	0.0019	0.0063	0.0000	0.0000	0.0193
Florida^d^	0.0017	0.0058	0.0000	0.0000	0.0177
Cyprus^d,f^	0.0017	0.0056	0.0000	0.0000	0.0159

^a^Bjorndal *et al.* 2006. ^b^Formia *et al.* 2007. ^c^Formia *et al.* 2006. ^d^Encalada *et al.* 1996. ^e^Bjorndal *et al.* 2005. ^f^Kaska 2000.
